# Travel-Related Malaria Diagnosis on Karius Test Despite Negative Blood Smear

**DOI:** 10.3390/tropicalmed10110310

**Published:** 2025-10-31

**Authors:** Joseph Eugene Weigold, Shankar Lal, Dima Ahmad Youssef

**Affiliations:** Division of Infectious Diseases, Department of Internal Medicine, East Tennessee State University, Johnson City, TN 37614, USA; weigoldj@mail.etsu.edu (J.E.W.); lals01@mail.etsu.edu (S.L.)

**Keywords:** *Plasmodium vivax*, travel-related malaria, Karius test, blood smear, febrile traveler, molecular diagnostics, malaria diagnosis, Amazon rainforest, non-endemic malaria, hypnozoite treatment

## Abstract

Malaria remains a considerable challenge to international health, especially in returning travelers from endemic regions where exposure risk may be downplayed. Prompt and accurate diagnosis is crucial, especially when conventional diagnostic techniques are insufficient. This case report presents a 59-year-old man who developed fever, rash, and myalgia after returning from the Amazon rainforest. Initial laboratory tests demonstrated leukopenia, thrombocytopenia, transaminitis, and hyperbilirubinemia. Despite these abnormal results and a clinically suspicious presentation, malaria smears were negative. Since the symptoms did not resolve, a Karius test—a plasma-based microbial cell-free DNA sequencing assay—successfully detected the presence of Plasmodium vivax, thus establishing the diagnosis. The patient needed several treatment regimens for the recurrent attacks, including chloroquine and primaquine, artemether-lumefantrine, and eventually a combination of quinine and doxycycline together with a prolonged course of primaquine. His symptoms resolved completely after the last treatment regimen, along with the normalization of the blood counts and liver function tests. This case demonstrates the limitations of smear microscopy diagnosis in *P. vivax* infections, highlights the role of molecular diagnostics like the Karius test, and stresses the importance of preventing relapses with adequate hypnozoite clearance. It further highlights the importance of clinician awareness and diligent follow-up in cases of travel-related Malaria, especially those with unusual presentations or recurrent symptoms.

## 1. Introduction

Malaria remains a significant infectious disease globally, infecting about 249 million people and killing over 608,000 people in 2022, mostly in Southeast Asian and sub-Saharan African countries [1]. Even though this disease is not endemic to the United States or other developed countries, the presence of imported Malaria from different parts of the world poses the challenge of diagnosis in non-endemic areas [2]. Travel-associated Malaria, particularly that caused by *Plasmodium vivax*, is anticipated to be underreported owing to its unpredictable incubation period, nonspecific symptoms, and low parasitemia levels, which lead to false-negative results when standard blood smear microscopy procedures are used [3]. The sensitivity of thick/thin smears declines below 50% in low-parasitemia *P. vivax* infections even though they remain to be the gold standard [4].

Traditional diagnostic methods, such as thick and thin blood smears, are still the gold standard for diagnosing Malaria; however, their sensitivity is operator-dependent and is significantly reduced in cases of low parasitemia [5]. Therefore, new diagnostic technologies, as represented by the Karius test—a metagenomic sequencing platform that can detect microbial cell-free DNA in plasma—have received attention because they can detect a greater variety of pathogens with increased sensitivity [6]. Several recent investigations have shown significant support to apply metagenomic next-generation sequencing, in short, mNGS, to detect the Plasmodium species among travelers. Importantly, this technique has sensitivities of more than 90 percent, which is significant, particularly when previous smears turn out to be negative [7].

The improvement of molecular diagnostic methods is particularly relevant within the context of *P. vivax*, which is responsible for dormant liver-stage hypnozoites that can re-emerge several weeks to months after the original illness. This condition tends to cause diagnostic uncertainties and delays in treatment [8]. A delayed or false diagnosis can cause unnecessary complications, including acute anemia, thrombocytopenia, and frequent relapses [9].

With increased international travel and improvements in diagnostic techniques, clinicians in areas where some diseases are not endemic must be aware of unusual clinical presentations and the limitations of the standard diagnostic methods. The case presentation in this article reports a patient who, after traveling to the Amazon basin, developed Malaria that standard microscopy could not detect but was correctly diagnosed with the use of the Karius test. The report highlights the role of new diagnostic tools in improving clinical decision-making and emphasizes the importance of careful evaluations of returning, febrile travelers from endemic regions.

## 2. Materials and Methods

### 2.1. Patient Evaluation and Ethical Approval

This research offers a thorough assessment of the evaluation and treatment of an adult patient who presented for care because of febrile illness after recent international travel. Since this is a retrospective case report without experimental interventions, ethical approval was not required per the institutional review board guidelines. The patient gave informed written consent to publish clinical results and findings under anonymous conditions, per the Declaration of Helsinki (2013 revision). No animal subjects were included in the study.

### 2.2. Clinical and Laboratory Workup

The initial evaluation included a thorough clinical history, noting recent travel to areas endemic for Malaria, the use of preventive measures, and possible environmental exposures. Physical examination findings, as well as vital signs, were recorded. Laboratory evaluations included a CBC, LFTs, and peripheral blood smear microscopy using Giemsa stain, performed according to the recommendations set by the CDC for diagnosing Malaria [5].

Given the patient’s recent travel to the Amazon state, both arboviral diseases and tick-borne diseases were included in the differential diagnosis. Serologic tests were ordered to identify the presence of Dengue, Zika, Oropouche, and Ehrlichia species. All tests were performed in a CLIA-certified laboratory.

### 2.3. Molecular Diagnostic Testing

As the fever persisted and routine diagnostic tests were non-conclusive, a Karius Test™ was ordered on Day 4 of admission. The Karius test is a metagenomic next-generation sequencing (mNGS) test that detects microbial cell-free DNA (mcfDNA) in plasma, allowing for comprehensive pathogen detection with excellent sensitivity [6,7]. The Karius test aims to recognize and identify different pathogens at extremely low concentrations, as low as 0.1 DNA molecules per microliter (μL). Additionally, it has also been noted and reported to be extremely sensitive to detecting Plasmodium, with a remarkable 92% sensitivity rate [7]. Plasma samples were collected in cfDNA-stabilizing tubes and then analyzed at Karius Inc. (Redwood City, CA, USA). The test result was positive for *Plasmodium vivax*, above the threshold of clinical reporting, which prompted the initiation of targeted antimalarial therapy.

### 2.4. Antimalarial Treatment

With molecular confirmation of *P. vivax*, treatment was started with oral chloroquine phosphate according to the dosing regimens recommended by the CDC (600 mg base, with 300 mg base at 6, 24, and 48 h). Because of the risk of hemolysis with primaquine therapy in patients with G6PD deficiency, G6PD testing was performed using a quantitative enzyme assay (Trinity Biotech, Bray, Ireland) [10]. After confirmation of a normal G6PD level, the patient started a 14-day course of primaquine (15 mg base per day), per the WHO recommendations for treating Malaria [11].

### 2.5. Use of Generative AI

The study design, data analysis, and scientific interpretation of findings did not involve any generative artificial intelligence (GenAI) software. However, some GenAI help, particularly Chrome 136.0.7091.2 Grammarly language editing and formatting software, was utilized in a way that prevented the scientific content from being altered.

## 3. Results

At admission, the patient had the following initial laboratory results: leukopenia, thrombocytopenia, and transaminitis. The values were as follows: WBC count 3.3 × 10^9^/L, hemoglobin 12.7 g/dL, platelet count 52,000/μL, creatinine 1.23 mg/dL, AST 105 U/L, ALT 124 U/L, alkaline phosphatase 190 U/L, and total bilirubin 2.3 mg/dL. These results indicate a systemic inflammatory response with liver involvement, a picture commonly seen in *Plasmodium vivax* infection [12].

A thorough serological workup was done to rule out differential diagnoses, all of which were negative. Tests ordered included HIV screening, acute hepatitis panel, dengue virus IgM and IgG serologies, dengue virus serum PCR, chikungunya virus serology, Zika virus serology, *Trypanosoma cruzi* serology, Oropouche virus serology, and a Lyme disease panel. Serologies for rickettsial diseases were also done; these were *Borrelia burgdorferi*, *Rickettsia rickettsii* (the agent of Rocky Mountain spotted fever), *Ehrlichia chaffeensis*, and *Anaplasma phagocytophilum*, all of which were negative as well. Two thick and thin blood smears for Malaria and Babesia were read, but no parasites were seen.

**Figure 1:**
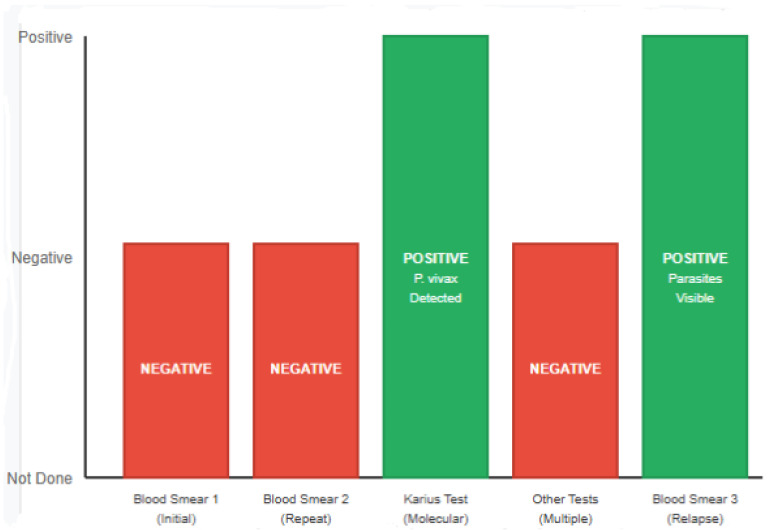
Clinical Timeline summarizing the patient’s diagnostic and therapeutic journey.

As symptoms persisted and standard diagnostic tests yielded inconclusive results, a Karius Test™ was performed, registering a positive result for *Plasmodium vivax*. Please refer to Figure 1 for a Clinical Timeline summarizing the patient’s diagnostic and therapeutic journey. Given this result, antimalarial treatment was initiated with chloroquine for three days, followed by primaquine (15 mg base) for fourteen days. The patient showed initial clinical improvement; however, weeks after the treatment, he developed a relapse of symptoms of lethargy, lightheadedness, fatigue, and fever—signs of a relapse of *P. vivax*, caused by the reactivation of liver-stage hypnozoites [13,14,15].

A second course of empirical treatment was administered via telemedicine, which consisted of artemether-lumefantrine (Coartem^®^, Sandoz, Basel, Switzerland) 20/120 mg—four tablets twice a day for 3 days—and primaquine 30 mg (26.3 mg salt) once a day for 14 days. Symptoms resolved on the second day of treatment. Nevertheless, the patient then relapsed, as evidenced by a positive malaria smear. Therefore, a more intense treatment regimen was started, which consisted of doxycycline 100 mg twice a day for 7 days, quinine sulfate 324 mg given as two tablets every 8 h for 3 days, and primaquine 30 mg (26.3 mg salt) once a day for 21 days.

Following administration of this third-line therapy, follow-up laboratory tests showed normalization of the parameters: WBC count was 5.9 × 10^9^/L, hemoglobin was 15.8 g/dL, platelet count was 165,000/μL, AST was 16 U/L, ALT was 23 U/L, alkaline phosphatase was 64 U/L, and total bilirubin was 0.5 mg/dL. The patient has remained asymptomatic, with no signs of relapse for more than six months. Please refer to Table 1 for summary of laboratory results over the course of the clinical course.

## 4. Discussion

This case identifies several essential difficulties in diagnosing and managing *Plasmodium vivax* malaria, especially in areas where it is not endemic. Even with classical clinical symptoms—fever, rash, and myalgia—standard blood smear microscopy did not reveal parasites on two occasions. This drawback of smear microscopy is widely recognized, particularly for *P. vivax*, which tends to have lower levels of peripheral parasitemia than *P. falciparum* [16]. In this context, an exclusive reliance on microscopy can lead to missed diagnoses, resulting in a need for increased clinical vigilance and the application of newer diagnostic methodologies in patients with appropriate travel histories.

The Karius test—a metagenomic cell-free DNA (mcfDNA) sequencing assay—was instrumental in revealing the etiologic pathogen when standard tests remained inconclusive. In contrast to smear microscopy or rapid diagnostic tests (RDTs), the Karius test is not reliant on detecting intact organisms; instead, it detects microbial DNA fragments, which allows for the identification of pathogens that are present in low titers or those that do not live in the bloodstream [6]. In this case, its role was not limited to diagnosis; it was also prognostic, as the early confirmation of *P. vivax* allowed for directed therapy and prevented additional diagnostic delays.

However, even following the correct initial treatment with chloroquine and primaquine, the patient experienced several relapses, each with similar clinical features and requiring increasingly aggressive treatment regimens. These relapses highlight the complex biology of *P. vivax*, particularly the presence of latent liver-stage hypnozoites that can recur weeks or even months after the primary infection [17]. Treatment failure, even with primaquine, can occur because of several reasons such as poor compliance, resistance to drugs, or reduced bioavailability [15].

In the last phase of treatment, a carefully tailored regimen of quinine, doxycycline, and a prolonged 21-day course of primaquine successfully relieved the symptoms. This highlights the need for clinicians to escalate treatment in cases of relapsing *P. vivax* malaria based on the patient’s history and relapse patterns. Importantly, the patient’s G6PD status was established before primaquine was started—a vital safeguard against the theoretical threat of hemolytic anemia associated with 8-aminoquinoline drugs [5].

From a public health point of view, this case reveals significant failings in travel health education. The patient used mosquito prophylaxis intermittently and chose not to use chemoprophylaxis, incorrectly assuming that a lack of seen mosquitoes meant low transmission risk. This widespread error in travelers highlights the importance of more detailed pre-travel consultation and enhanced risk communication about vector-borne diseases [18].

Moving forward, future research should evaluate the utility of mcfDNA sequencing in larger populations, particularly for malaria subtypes prone to diagnostic escape. The development of rapid, reliable G6PD testing at the point of care is also critical to safely administering primaquine and tafenoquine in resource-limited or time-sensitive settings. Moreover, strengthening guidelines for relapse management in *P. vivax* malaria will help clinicians navigate complex cases involving therapeutic failure or drug resistance. These relapses can also be due to inadequate clearance of hypnozoites, something which can happen due to abbreviated doses, as noted by the World Health Organization in 2022 [11].

This case highlights the importance of combining clinical acumen with state-of-the-art diagnostic technology for travelers presenting with fever. It also highlights the need for long-term, especially in the event of relapsing Malaria, and promotes the increased use of next-generation sequencing methods within infectious disease diagnosis [6]. In addition, public health initiatives must target providing advice prior to travel. In 2022, only 40% of travelers to disease-endemic areas received prevention counseling consistent with our patient’s experience. Physicians should adhere to CDC/WHO recommendations during consultations.

## 5. Conclusions

This case helps to highlight the complex and often complicated diagnostic nuances involved in *Plasmodium vivax* malaria, mainly when it occurs in non-endemic areas where the disease is not regularly encountered. This concern is even more significant when conventional diagnostic techniques, like microscopy, fail and return false-negative results, which can result in misdiagnosis and, subsequently, delayed treatment. The fact that the pathogen was successfully identified using the novel Karius test is a compelling example of the vital contribution made by cutting-edge molecular diagnostics. Next-generation sequencing and related techniques are essential in accurately detecting those cryptic pathogens that may otherwise remain undetected and, as such, facilitate timely and specific treatment strategies for the patient. Clinicians must have a high index of clinical suspicion for the potential of Malaria in returning travelers presenting with unexplained fever-like symptoms, even in cases where the initial diagnostic tests prove to be negative. The early and timely institution of radical cure regimens is of the utmost importance in preventing any future relapse of the condition. The widespread availability of these cutting-edge molecular diagnostic instruments, in conjunction with enhanced pre-travel prophylactic practices, is the key and necessary measure to effectively curb the incidence and the overall burden of imported malaria cases.

## Figures and Tables

**Table 1:** Summary of laboratory results over the clinical course.

Parameter	Normal Range	At Admission	After the First Relapse	Final Follow-Up
WBC (×10^9^/L)	4.0–11.0	3.3	N/A	5.9
Hemoglobin (g/dL)	13.5–17.5 (male)	12.7	N/A	15.8
Platelets (/μL)	150,000–450,000	52,000	N/A	165,000
Creatinine (mg/dL)	0.6–1.3	1.23	N/A	N/A
AST (U/L)	10–40	105	N/A	16
ALT (U/L)	7–56	124	N/A	23
Alkaline Phosphatase (U/L)	44–147	190	N/A	64
Total Bilirubin (mg/dL)	0.1–1.2	2.3	N/A	0.5
Malaria Smear	Negative	Negative ×2	Positive	Negative
Karius Test	N/A	Positive (*P. vivax*)	N/A	N/A

N/A = not available.

## Data Availability

The original contributions presented in this study are included in the article/Appendix A. Further inquiries can be directed to the corresponding author.

## Abbreviations

The following abbreviations are used in this manuscript:

ALT	Alanine Aminotransferase
AST	Aspartate Aminotransferase
CBC	Complete Blood Count
CDC	Centers for Disease Control and Prevention
cfDNA	Cell-Free DNA
CLIA	Clinical Laboratory Improvement Amendments
DNA	Deoxyribonucleic Acid
G6PD	Glucose-6-Phosphate Dehydrogenase
LFT	Liver Function Test
mcfDNA	Microbial Cell-Free DNA
mNGS	Metagenomic Next-Generation Sequencing
PCR	Polymerase Chain Reaction
RDT	Rapid Diagnostic Test
WBC	White Blood Cell Count
WHO	World Health Organization

## Appendix A

Table A1 presents a summary of the patient’s abnormal laboratory values recorded throughout the course of illness and recovery. These values, highlighted in the patient’s electronic health records, reflect the progression and resolution of malaria-related hematological and hepatic abnormalities. The table includes key deviations from reference ranges, such as leukopenia, thrombocytopenia, elevated liver enzymes, and hyperbilirubinemia, which are commonly associated with *Plasmodium vivax* infection. Tracking these parameters provided crucial insight into disease activity, therapeutic response, and relapse events. This detailed timeline supports the clinical narrative and emphasizes the need for vigilant laboratory monitoring in cases of recurrent or relapsing Malaria.

**Table A1:** Highlighted laboratory abnormalities over time.

Date	Test	Value	Reference Range
08/27/2024	Glucose	118 mg/dL	74–106 mg/dL
08/27/2024	AST	105 U/L	13–40 U/L
08/27/2024	ALT	124 U/L	10–49 U/L
08/27/2024	Alkaline Phosphatase	190 U/L	46–116 U/L
08/27/2024	Total Bilirubin	2.3 mg/dL	0.2–1.2 mg/dL
08/28/2024	AST	126 U/L	13–40 U/L
08/28/2024	ALT	126 U/L	10–49 U/L
08/28/2024	Alkaline Phosphatase	149 U/L	46–116 U/L
08/28/2024	Total Bilirubin	1.5 mg/dL	0.2–1.2 mg/dL
08/30/2024	AST	66 U/L	13–40 U/L
08/30/2024	ALT	137 U/L	10–49 U/L
08/30/2024	Alkaline Phosphatase	205 U/L	46–116 U/L
08/30/2024	Total Bilirubin	1.4 mg/dL	0.2–1.2 mg/dL
11/12/2024	Total Bilirubin	1.5 mg/dL	0.2–1.2 mg/dL
01/24/2025	Total Bilirubin	1.5 mg/dL	0.2–1.2 mg/dL
08/27/2024	WBC	3.3 × 10^3^/μL	4.10–10.00 × 10^3^/μL
08/27/2024	Platelet Count	52 × 10^3^/μL	150–400 × 10^3^/μL
08/27/2024	Hemoglobin	12.7 g/dL	13.5–17.8 g/dL
11/12/2024	Lymphocytes %	8.9%	17.0–48.0%
11/12/2024	Monocytes %	2.4%	4.0–13.0%
01/24/2025	Eosinophils #	0.11 × 10^3^/μL	0.00–0.80 × 10^3^/μL
01/24/2025	Monocytes #	0.41 × 10^3^/μL	0.20–1.20 × 10^3^/μL
11/12/2024	Immature Granulocytes %	1.3%	0.0–0.5%
11/12/2024	Immature Granulocytes #	0.05 × 10^3^/μL	0.00–0.30 × 10^3^/μL

Note: %: percentage; #: absolute count.
